# Optimal time for MRI response evaluation in squamous cell carcinoma of the anus

**DOI:** 10.1093/bjro/tzag004

**Published:** 2026-02-11

**Authors:** Bettina A Hanekamp, Ellen Viktil, Johann Baptist Dormagen, Nils E Kløw, Cathrine Brunborg, Eirik Malinen, Marianne G Guren, Anselm Schulz

**Affiliations:** Institute of Clinical Medicine, University of Oslo, 0318 Oslo, Norway; Department of Radiology, Oslo University Hospital, 0424 Oslo, Norway; Institute of Clinical Medicine, University of Oslo, 0318 Oslo, Norway; Department of Radiology, Oslo University Hospital, 0424 Oslo, Norway; Department of Radiology, Oslo University Hospital, 0424 Oslo, Norway; Institute of Clinical Medicine, University of Oslo, 0318 Oslo, Norway; Department of Radiology, Oslo University Hospital, 0424 Oslo, Norway; Oslo Centre for Biostatistics and Epidemiology, Oslo University Hospital, 0424 Oslo, Norway; Department of Physics, University of Oslo, 0316 Oslo, Norway; Department of Radiation Biology, Oslo University Hospital, 0310 Oslo, Norway; Institute of Clinical Medicine, University of Oslo, 0318 Oslo, Norway; Department of Oncology, Oslo University Hospital, 0310 Oslo, Norway; Institute of Clinical Medicine, University of Oslo, 0318 Oslo, Norway; Department of Radiology, Oslo University Hospital, 0424 Oslo, Norway

**Keywords:** anal cancer, diffusion magnetic resonance imaging, response assessment, chemoradiotherapy, treatment failure

## Abstract

**Objectives:**

To identify the optimal time for MRI response evaluation after chemoradiotherapy (CRT) in squamous cell carcinoma of the anus (SCCA) and to employ combined T2+diffusion-weighted MRI tumour regression grade (comrTRG).

**Methods:**

We assessed the positive and negative predictive values (PPV, NPV) of post-treatment MRI in a retrospective mono-centre diagnostic accuracy study that prospectively included consecutive patients treated between 2013 and 2017. Index tests were MRI at 6-, 12-, and 24-weeks post-treatment (6w, 12w, and 24w) to detect locoregional treatment failures (LRTF). Clinical outcome served as reference standard. Tumour regression was assessed using comrTRG based on radiological reports. Mixed-effects logistic regression was used to compare the comrTRG score across time points. The analyses were stratified by patients’ T/N stage and human papillomavirus (HPV) status.

**Results:**

For 127 included patients (62 years ± 11 [mean ± SD]; 92 women), 261 post-treatment MRI reports (6w: *n* = 45, 12w: *n* = 125, 24w: *n* = 91) were scored using comrTRG. LRTF occurred in 13 patients; 12/13 were high-risk patients (T3/T4, N+, or HPV-negative); 1/13 progressed early (<24 weeks). The rate of radiologic complete response (comrTRG1) increased over time (6w: 27%, 12w: 66%, 24w: 75%), while the rate of indeterminate (comrTRG2) and minor definite tumour (comrTRG3) decreased. PPV of MRI increased over time: 6w: 33% (95%CI: 9.9%-65.1%), 12w: 46% (16.7%-76.6%), and 24w: 88% (47.3%-99.7%). NPV was stable high >90%.

**Conclusions:**

MRI performed more reliably after 24 weeks. Timely assessment may aid early LRTF detection. Tailoring follow-up with frequent MRI scans may be sufficient for high-risk patients. Combined mrTRG is practical for describing response in SCCA.

**Advances in knowledge:**

Post-treatment MRI assessment at later time points is preferable in SCCA to avoid inconclusive imaging and unnecessary salvage surgery. The introduced comrTRG is a practical tool for response evaluation.

**Registration:**

ClinicalTrials.gov: NCT01937780.

## Introduction

Squamous cell carcinoma of the anus (SCCA) is treated with curative intent with chemoradiotherapy (CRT) with mitomycin (MMC) and 5-fluorouracil (5-FU) or capecitabine.^1–4^ The overall survival rate after treatment has improved over the last decades; however, patients with locally advanced disease still have a worse outcome.^2^ Highest relapse rate and poorest survival rate are shown for patients with T3-4N+ disease.^5^ Additionally, factors related to human papillomavirus (HPV) negative status (HPV, p16, and p53) are associated with worse survival outcomes.^6^ For patients with local residual or recurrent disease 6 months after CRT, curatively intended salvage surgery, often involving extensive pelvic procedures,^7^ is the preferred treatment. Close monitoring for persistent disease is recommended for up to 6 months following CRT.^8–10^

Response after CRT is traditionally assessed clinically, and the recommended time point for response assessment varies from 6 to 12 weeks after treatment completion.^8^^,^^10^ A recent publication showed that many patients achieve complete response up to 26 weeks from the start of CRT, suggesting that 26 weeks is the best time to assess clinical response.^3^^,^^9^ Clinical response evaluation is often accompanied by magnetic resonance imaging (MRI).^3^^,^^11^ However, there is little knowledge about the best time point. Importantly, consensus criteria or recommendations for the MRI protocol and optimal time point do not exist.^12^^,^^13^ In many centres, MRI is performed 6-8 weeks post-treatment, as traditionally in rectal cancer, which we assume to be too early. Post-treatment pelvic MRI in SCCA typically shows an absent or shrunken tumour and a reduction of the T2 signal in the tumour area, likely representing fibrosis. Diffusion-weighted imaging (DWI) can distinguish tumour from post-treatment changes by demonstrating restricted tumour diffusion^14^^,^^15^ and is an inherent part of our imaging protocol. The tumour regression grade (TRG) is a score used by pathologists to evaluate the effect of neoadjuvant treatment on surgically resected tumours.^16^ In rectal cancer, an MRI-based TRG is established to assess response after CRT. By adding DWI to traditional T2WI, a superior performance is reported^17–20^ and has recently been confirmed in SCCA.^21^ Post-treatment MRI at the optimal time can accurately identify locoregional failures, reducing inconclusive results and unnecessary surgeries.

This study aimed to explore the most reliable time point for MRI-based response assessment after CRT in patients with SSCA by evaluating the diagnostic accuracy of MRI at 6, 12, and 24 weeks post-treatment to detect locoregional treatment failure (LRTF). Secondary aims were to translate and combine T2WI and DWI patterns described in radiological reports in patients with SCCA into a novel single score called comrTRG, and to examine the feasibility of an individualized follow-up strategy based on patient-specific risk patterns (T/N stage and HPV status).

## Methods

The presented data in this study follow the STARD 2015 guidelines for reporting diagnostic accuracy studies (REF: https://doi.org/10.1148/radiol.2015151516).

### Study design and patient population

This is a retrospective diagnostic accuracy study of a prospective cohort. It is part of the prospective multidisciplinary observational “Anal Cancer Radiotherapy—Prospective study of treatment outcome, patient-reported outcomes, utility of imaging and biomarkers, and cancer survivorship (ANCARAD)” study (NCT01937780). Main inclusion criteria have previously been reported^22^^,^^23^ and, in brief, were histologically proven SCCA, planned CRT, and adequate performance status. All patients provided written informed consent. The study was approved by the Regional Ethical Committee South–East (2012/2274) and the local data protection officer. A total of 132 of 140 patients with histologically proven SCCA referred to Oslo University Hospital (OUS) between October 2013 and September 2017 received CRT and were consecutively included. ANCARAD patient baseline demographic and clinical characteristics have previously been reported.^22^ According to prior studies^5^^,^^6^^,^^22^ patients with advanced tumours, lymph node metastasis, or a negative HPV status (T3/T4, N+, or HPV−) were considered as the high-risk cancer group as opposed to the low-risk cancer group (T1/T2, N−, or HPV+). If not contraindicated, patients underwent pelvic MRI before and at several timepoints after CRT for staging, response evaluation, and follow-up.

### Chemoradiotherapy, response evaluation, and follow-up

Patients were treated according to national guidelines after being discussed in a multidisciplinary team meeting. Standard radiotherapy was delivered using 3D conformal radiotherapy, intensity-modulated radiotherapy, or volumetric modulated arc therapy (VMAT). Depending on stage, the administered radiotherapy doses to the primary tumour and the metastatic lymph nodes were 54 or 58 Gy; the administered dose to the elective nodal regions was 46 Gy. Patients received chemotherapy with MMC 10 mg/m^2^/day on day 1 and chemotherapy with 5-FU 1000 mg/m^2^/day on days 1-4. A new cycle of MMC/5-FU was delivered on day 29 for patients with locally advanced disease. Further treatment details were published previously.^22^

Tumour response was routinely assessed 12 weeks after CRT by clinical examination, including anoscopy/proctoscopy and pelvic MRI. All patients were followed for at least 5 years or until death or recurrence. Patients who had residual disease or later developed recurrence were considered for salvage surgery.

### Clinical outcome and reference standard

The primary clinical outcome in the present study was LRTF. It was defined as a lack of complete response 6 months after CRT or evidence of local or regional disease after complete response had been achieved, confirmed by pathology. This definition served as the reference standard.

### MRI

All included patients underwent pelvic MRI at 12 weeks and/or 24 weeks after CRT, but a subgroup had an additional pelvic MRI at 6 weeks after CRT, following a former routine (6w, 12w, and 24w). The index test was MRI at the 3 time points. The scans were performed using various 1.5T or 3T MRI scanners, with an acquisition protocol that included a combination of high-resolution T2WI and DWI, covering at least 2 b-values (b0 and b1200/b1000). Patients were scanned in the supine position using a pelvic phased array coil. To reduce bowel movement artefacts, patients received 1 mg glucagon (Glucagon^®^, Novo Nordisk) intramuscularly and 20 mg butylscopolamine (Buscopan^®^, Sanofi-Aventis) intravenously, before and during scanning, if not contraindicated.

### Combined MR tumour regression grade (comrTRG)

To assess tumour response, radiology reports for the MRI examinations performed at 6, 12, and 24 weeks after completion of CRT were extracted from the radiology information system. At our institution, the report typically represents a consensus reading of 2 radiologists, at least one of whom has more than 5 years of experience in reporting abdominal and pelvic MRIs. The appearance of the tumour on T2WI and DWI was described in the radiologic routine report, though the use of a TRG scale was not part of the report. For this study, a board-certified radiologist (B.A.H.) with extensive experience in pelvic MRI (>15 years) retrospectively translated the described regression of the tumour in the given reports and graded it using a 5-point comrTRG (Table 1). The introduced comrTRG reflects the literature on this topic,^24–26^ and the clinical routine at our institution. No other clinical information or the outcome (reference standard) was available in this setting.

**Table 1.** Description of imaging findings in tumour regression grade based on T2+diffusion-weighted MRI (comrTRG); score 1-5.

Grade	Imaging findings	T2WI	DWI
comrTRG 1	Complete response No tumour	Normal wall or thin fibrosis	No foci of high signal on b1000-b1200
comrTRG 2	Near-complete response Indeterminate tumour	Normal wall or thin fibrosis	Few minimal diffuse areas of high signal on b1000-b1200
comrTRG 3	Moderate response Minor definite tumour in predominant fibrosis (< 50%)	Normal wall or thin fibrosis or Small area with high T2 signal	Small measurable area of high signal on b1000- b1200 and low signal on ADC map
comrTRG 4	Poor response Major definite tumour and little fibrosis (> 50%)	Predominant area with high T2 signal	Predominant area with high signal on b1000- b1200 and low signal on ADC map
comrTRG 5	No response or progression	Same appearance as pre-treatment or progression, no fibrosis evident	Same appearance as pre-treatment or progression

Abbreviations: ADC = apparent diffusion coefficient; DWI = diffusion-weighted imaging; T2WI = T2-weighted imaging.

### Statistical analysis

All statistical analyses were performed by B.A.H. and A.S., consulted by a senior statistician (C.B.), and using STATA (Statistical Software: Release 17, College Station, TX, United States: Stata Corp LLC). *Longitudinal changes:* For descriptive statistics, means with standard deviation (SD) or percentages are presented. Mixed-effect logistic regression was used to compare the comrTRG score distribution at the 3 time points and to account for repeated measures. Time and risk status were introduced as fixed effects, and a random intercept was used. Confidence intervals (CIs) were given as 95% CI, and *P*-values < .05 were considered significant.

*Diagnostic performance:* We estimated the diagnostic performance of MRI in detecting LRTF at the 3 time points (6w, 12w, and 24w) using different diagnostic measures (sensitivity, specificity, positive predictive value [PPV], and negative predictive value [NPV]). Clinical outcome (LRTF) during the follow-up interval served as reference standard. We defined the cutoff between comrTRG2 and comrTRG3 as the cutpoint for radiologic LRTF and compared its performance with that of the cutpoint between comrTRG1 and comrTRG2. PPV and NPV were defined as the most relevant measures for the diagnostic performance of MRI. All the above statistics were calculated for the entire patient group and then within the strata of the high-risk and low-risk groups. However, given the low number of events within the low-risk group, subgroup comparisons were reported descriptively with 95% CIs without formal hypothesis testing. Not all patients underwent MRI at all 3 time points, missing examinations were considered missing at random (MAR). Patients with missing data (MAR) on the reference standard and the index test were excluded from the analysis. All eligible patients in the ANCARAD study were included.

## Results

### Study design and patient population

**Figure 1.**
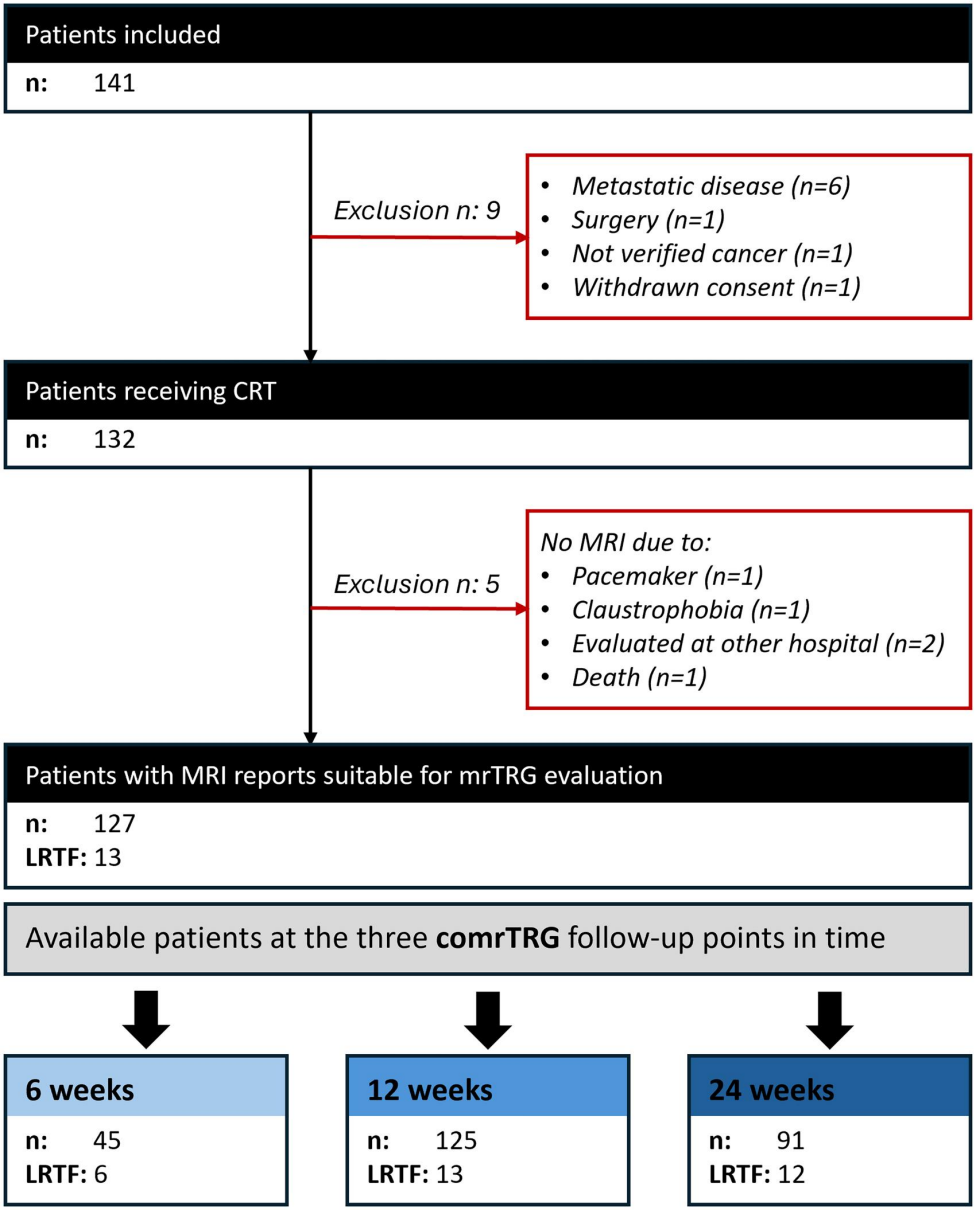
Flowchart showing the inclusion and exclusion of patients in the ANCARAD study and for the present MRI analyses. Abbreviations: comrTRG = combined MR tumour regression grade; CRT = chemoradiotherapy; LRTF = locoregional treatment failure.

The final cohort consists of 127 SCCA patients who received CRT with post-treatment MRI reports suitable for comrTRG evaluation. Almost all included patients (125/127) had an MRI scan for response evaluation 12 weeks after completing treatment. A subgroup of 45 patients received a scan after 6 weeks, while 91 patients had an MRI scan after 24 weeks. Figure 1 presents a flowchart illustrating the inclusion and exclusion criteria for patients. A summary of patients and tumour characteristics is shown in Table 2 and aligns closely with the data from the main study.^22^

**Table 2.** Summary of patient and tumour characteristics.

Characteristic	Patients (*n* = 127)
Age (years)	62 ± 11
**Sex**	
Female	92 (72%)
Male	35 (28%)
**T-stage (TNM 7)**	
T1	13 (10%)
T2	62 (49%)
T3	23 (18%)
T4	29 (23%)
**N-stage**	
N−	70 (55%)
N+	57 (45%)
**HPV− status**	
HPV−	23 (18%)
HPV+	103 (82%)
**High-risk group**	
T3/T4, N+ or HPV−	83 (66%)
**Low-risk group**	
T1/T2, N− or HPV+	43 (34%)

Values are presented as mean ± standard deviation or as the number of participants with the percentage of total in parentheses.

Abbreviation: HPV = human papilloma virus.

### Clinical outcome and reference standard

LRTF occurred in 13/127 (10%) patients. Of these, 8 patients had residual tumour after 6 months, and 5 patients developed a recurrence after complete response had been achieved. For the latter, the median time to locoregional recurrence/regrowth from the start of CRT was 76 weeks (range 64-188 weeks). Almost all (12/13) LRTFs occurred in the high-risk group. None of the 8 patients with locoregional residual tumour underwent salvage surgery less than 24 weeks after CRT. However, one high-risk patient had an early progression of a residual tumour and a new lung metastasis at 12 weeks and received chemotherapy before salvage surgery.

### Diagnostic performance

**Figure 2.**
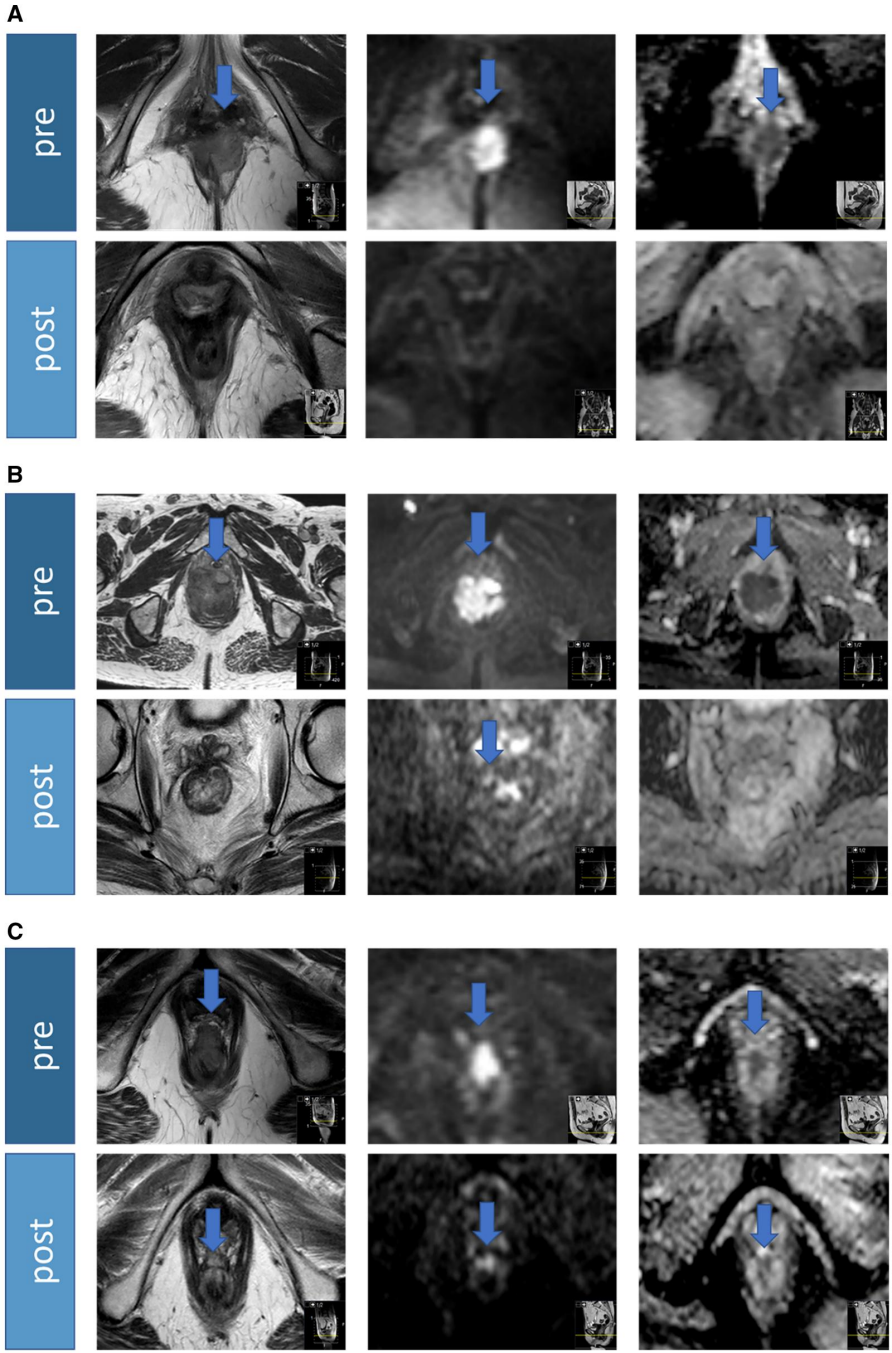
Imaging examples of tumour regression grade based on T2+diffusion weighted MRI (comrTRG) score 1-3. Images show transversal T2WI (left), DWI b800 (mid), and ADC (right) before and after treatment with CRT. (A) comrTRG1: complete response/no tumour, (B) comrTRG2: near-complete response/indeterminate tumour, and (C) comrTRG3: moderate respons/minor definite tumour in predominant fibrosis. Blue arrows mark the tumor. Abbreviations: comrTRG = combined MR tumour regression grade; ADC = apparent diffusion coefficient; DWI = diffusion-weighted imaging; T2WI = T2-weighted imaging.

A total of 261 MRI reports were analysed. The described tumour regression in the given reports was graded according to comrTRG (Table 1). Imaging examples of comrTRG1, comrTRG2, and comrTRG3 are shown in Figure 2.

**Figure 3.**
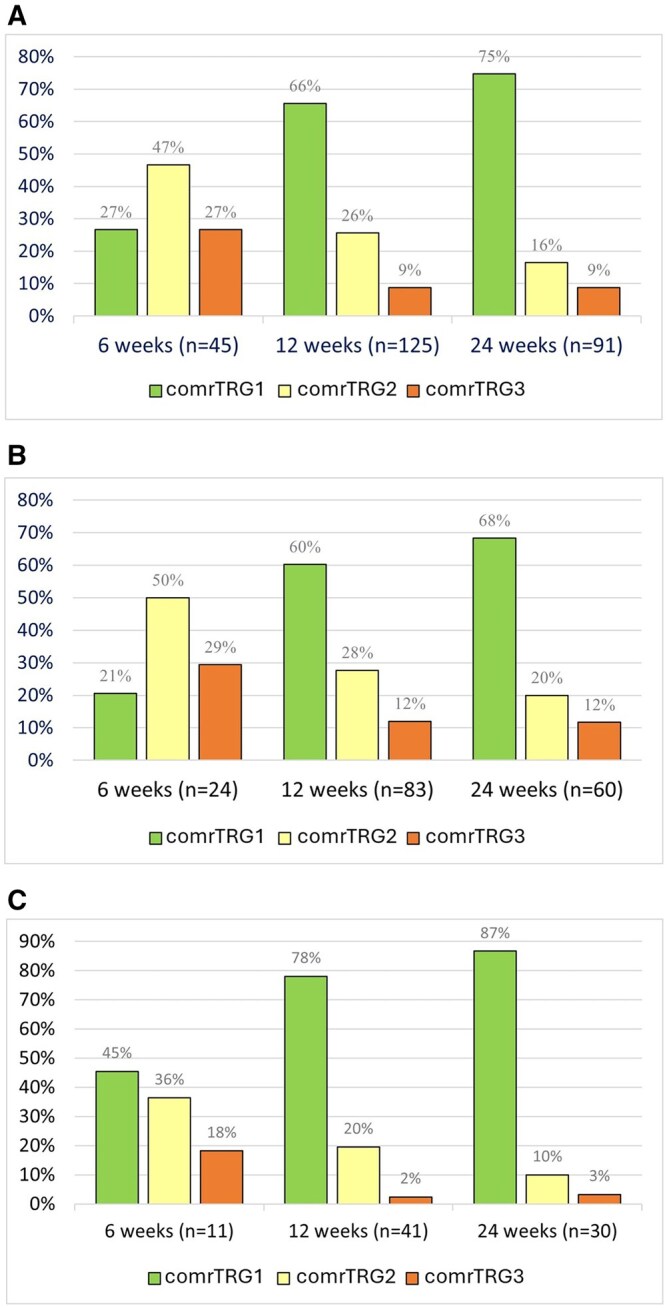
Distribution of tumour regression grade based on T2+diffusion weighted MRI (comrTRG) comrTRG score 1-3 on MRI 6, 12, and 24 weeks after completing CRT. (A) comrTRG score distribution for all included patients. (B) comrTRG score distribution for the high-risk patient group (*n* = 83/127): T3/T4, N+ or HPV−. (C) comrTRG score distribution for the low-risk patient group (*n* = 43/127): T1/T2, N− or HPV+. Abbreviations: comrTRG = combined MR tumour regression grade; HPV = human papillomavirus.

The rate of comrTRG1 increased with elapsed time from CRT and was significantly higher at 12 and 24 weeks compared to 6 weeks (*P* < .001). The corresponding rates of comrTRG2 (12 weeks, *P* = .009 and 24 weeks, *P* < .001) and comrTRG3 (*P* = .003, *P* = .002) decreased significantly with time after CRT (Figure 3A). Although the rate of comrTRG1 at 6 weeks appeared higher in the low-risk group (45%) than in the high-risk group (21%), this difference should be interpreted descriptively due to the small subgroup sizes and very few events (Figure 3B and C). The observed trends in the overall analysis were unchanged when stratified by risk status for all TRG levels; however, the subgroup sample size was low.

With the cutpoint between comrTRG2 and comrTRG3, the diagnostic performance of MRI was best after 24w. PPV increased with elapsed time and was highest at 24 weeks with 87.5% (47.3%-99.7%), while NPV was > 90% on all 3 time points (Table 3). The diagnostic performance was very good for the low-risk group 24 weeks post-treatment with PPV & NPV of 100% (CI PPV 2.5%-100%, NPV 88.1%-100%). However, CIs were broad due to the small number of patients in the group, and only one LRTF occurred (Tables A and B in the Supplementary Material). Considering the cutpoint between comrTRG1 and comrTRG2 and comparing it with LRTF, the diagnostic performance changed towards higher sensitivity and lower specificity; PPV decreased to 26% after 12 weeks and 36% after 24 weeks, though NPV remained >90% (Tables C-E in Supplementary Material).

**Table 3.** All included patients: diagnostic performance of MRI 6, 12, and 24 weeks after completion of chemoradiotherapy to detect locoregional treatment failure (LRTF), with the cutpoint between combined MR tumour regression grade 2 (comrTRG2) and grade 3 (comrTRG3).

	6 weeks	12 weeks	24 weeks
Total (*n*)	45	125	91
LRTF (number)	6	13	12
Sensitivity (95% CI) (%)	66.7 (22.3-95.7)	38.5 (13.9-68.4)	58.3 (27.7-84.8)
Specificity (95% CI) (%)	79.5 (63.5-90.7)	94.6 (88.7-98.0)	98.7 (93.1-100.0)
PPV (95% CI) (%)	33.3 (9.9-65.1)	45.5 (16.7-76.6)	87.5 (47.3-99.7)
NPV (95% CI) (%)	94.9 (80.0-99.3)	93.0 (86.6-96.9)	94.0 (86.5-98.0)

Clinical outcome (LRTF) served as the reference standard.

Abbreviations: CI = confidence interval; LRTF = locoregional treatment failure; NPV = negative predictive value; PPV = positive predictive value.

## Discussion

By assessing post-treatment tumour regression on MRI reports, we found that the rate of radiologic complete response (comrTRG1) increased with elapsed time from CRT. In contrast, the rate of indeterminate or minor definite tumours (comrTRG2 and comrTRG3) decreased. Using the cutoff between comrTRG2 and comrTRG3 as the diagnostic cutpoint, we found that the diagnostic performance of post-treatment MRI scans in detecting LRTF increased considerably with elapsed time from CRT and was best after 24 weeks. We found no advantage in using the cutoff between mrTRG1 and mrTRG2, as it reduced MRI’s performance in detecting LRTF, despite a slight increase in sensitivity with time. These results align with our current clinical practice. Almost all LRTF cases in our study occurred in the high-risk group, and individualized follow-up with frequent MRI scans, specifically for high-risk patients (T3/T4, N+, or HPV negativity), may be an option.

Currently, no consensus criteria or guidelines exist regarding the optimal time point to evaluate response in SCCA on MRI. The literature on MRI response evaluation in SCCA is scarce, comprising a few review articles^12^^,^^14^^,^^27^ and some recent studies.^21^^,^^28–30^ These are small studies that do not firmly distinguish between response evaluation at 3 and 6 months, and 2 of the studies do not even involve DWI.^28^^,^^29^ One common feature is that they mainly favour MRI response evaluation at time points later than 6-8 weeks after treatment. This aligns with our finding that the diagnostic performance of MRI improved over time and was best after 24 weeks. We found that the assessment at earlier time points was associated with higher rates of indeterminate or minor definite tumour (comrTRG2 or comrTRG3), likely representing both a lack of response and false-positive findings. Evaluation after 6 weeks showed the lowest proportion of radiological complete response (comrTRG1). This time point is considered too early, as patients with comrTRG2 or comrTRG3 could still achieve a complete response with time. Besides the post-treatment shrinkage of the tumour and lymph node metastases, a wide range of changes was observed on post-treatment MRI scans. For instance, the emergence of areas of low signal intensity in the former tumour area represents fibrosis/scar tissue, and diffuse areas of increased T2 signal intensity are due to inflammatory changes.^12^^,^^14^^,^^29^^,^^31^ These nonspecific, treatment-induced tissue alterations challenge the evaluation of early MRI responses. They may be the primary reasons for false-positive findings and low specificity, as observed in our study. Using data from the ACT II clinical trial, Glynne-Jones et al^9^ suggest that 26 weeks after the start of CRT (equivalent to 21 weeks after completion of CRT) is the best time to assess clinical response in SCCA, which aligns with our findings. In our opinion, it remains essential to consider earlier imaging evaluation, such as after 12 weeks, especially for patients with clinically uncertain findings, due to the risk of early progression of local residual tumour and metastatic spread, as observed in one patient in our study.

DWI can distinguish between tumour and post-treatment changes by demonstrating restricted tumour diffusion,^14^^,^^15^ and is an integral part of our MRI protocol. We introduced novel combined mrTRG, based on DWI + T2WI: comrTRG. In the literature, various MRI-TRG systems have been used for response evaluation in rectal cancer and SCCA. Some were based solely on T2WI^28^^,^^32^^,^^33^; later studies described a superior diagnostic performance for adding DWI to T2WI^17^^,^^18^^,^^20^^,^^25^^,^^26^^,^^34^ in rectal cancer, which has recently been confirmed in SCCA.^21^ The applied MRI TRG systems employ different MRI protocols and define slightly different grades, with no consensus or guidelines in place. We believe that our combined comrTRG provides an accurate assessment of the challenges and requirements in evaluating response in SCCA, and this work presents the first use of our scoring system, showing its practicability.

The clinical outcome in our study was LRTF, defined as a lack of complete response 6 months after CRT or evidence of local or regional disease recurrence after achieving a complete response. Almost all LRTFs occurred in the high-risk group; only one patient in the low-risk group had an LRTF (residual tumour). Notably, this patient had some other characteristics described in the literature as potential risk factors, such as male gender, perianal fistula, and tumour size exceeding 4 cm, which should increase alertness. Risk-adapted MRI follow-up may be a logical approach, with frequent MRI in high-risk patients and less frequent or no MRI in the follow-up of low-risk patients. We have already implemented this approach in our national guidelines, expecting it to result in more restrictive use of MRI in the follow-up of patients with SCCA, thereby reducing the burden on patients and the costs to the healthcare system. In SCCA, the percentage of LRTF declined to approximately 17% between 2010 and 2014.^35^^,^^36^ In a study by Sehkar et al, the models predicted a 3-year LRTF of 14.7% for 2020. Besides the described decline in LRTF, we assume that the even lower rate of LRTF in our study may be a random event related to the relatively small number of patients included in our single-centre study.

The main strengths of our study are the inclusion of a large patient number, the routine involvement of DWI in response evaluation in SCCA, and the detailed assessment of the accuracy of post-treatment MRI at 3 time points. Limitations exist despite the relatively large number of included patients; however, the numbers in the subgroups and the number of LRTF are still low, which limits the statistical power and interpretability. Validation in studies with a larger number of patients and LRTFs, potentially from multicentre studies, is needed. A feasible approach could involve the use of distributed learning.^37^ The radiology report used to assess treatment response represents a consensus reading of 2 radiologists; however, there may still be a risk of confirmation bias, as only one radiologist (B.A.H.) translated the reports from the radiology information system into comrTRG. Prior beliefs or experiences could have affected the translation. The information needed for the comrTRG score is part of our routine MRI reports. Therefore, we believe this potential bias did not significantly affect the results, as the conversion for study purposes was intuitive and straightforward.

Additionally, using clinical MRI reports for this analysis is challenging, as probably other clinical or imaging information may have influenced the report and scoring. We encourage future research to use the introduced comrTRG upfront by independent readers and to test inter- and intraobserver agreement on blinded imaging data. Finally, not all patients underwent MRI scans at all 3 time points (6, 12, and 24 weeks), and there may be a selection bias in the choice of time points. The number of MRI scans after 24 weeks (*n* = 91) was lower than after 12 weeks (*n* = 125), most likely because a complete response was already diagnosed after 12 weeks and a new scan was therefore omitted after 24 weeks. Having this in mind, the diagnostic performance of MRI after 24 weeks would have been even better if these patients had been scanned at this time point as well. In general, the relatively high amount of missing data should be considered a weakness of our study, emphasizing its exploratory nature.

In conclusion, the diagnostic performance of post-treatment MRI was more reliable after 24 weeks. Timely assessment, such as at 12 weeks, may assist in detecting early LRTF, especially when clinical findings are uncertain. Since almost all LRTF occurred in the high-risk group, individualizing follow-up with frequent MRI scans solely for high-risk patients (T3/T4, N+, or HPV negativity) might be an option. The introduced combined comrTRG is practical for describing responses in patients with SCCA.

## Supplementary Material

tzag004_Supplementary_Data
