# Supplementary material for: Optimal time for MRI response evaluation in squamous cell carcinoma of the anus
Source: BJR Open. 2026 Feb 11;8(1):tzag004. doi: 10.1093/bjro/tzag004 (PMC12947585; doi:10.1093/bjro/tzag004)
Supplement: tzag004_Supplementary_Data [file tzag004_supplementary_data.docx]

**Supplementary material:**

Table A*. High-risk patient group*: diagnostic performance of MRI 6 weeks, 12 weeks, and 24 weeks after completion of chemoradiotherapy to detect locoregional treatment failure (LRTF), with the cutpoint between combined MR tumor regression grade 2 (comrTRG2) and grade 3 (comrTRG3). Clinical outcome (LRTF) served as the reference standard.

|  | **6 weeks** | **12 weeks** | **24 weeks** |
| --- | --- | --- | --- |
| Total (n) | 34 | 83 | 60 |
| LRTF (number) | 6 | 12 | 11 |
| Sensitivity (95 % CI) | 66.7 (22.3 - 95.7) | 41.7 (15.2 -72.3) | 54.5 (23.4 - 83.3) |
| Specificity (95% CI) | 78.6 (59.0 - 91.7) | 93.0 (84.3 - 97.7) | 98.0 (89.1 - 99.9) |
| PPV (95% CI) | 40.0 (12.2 - 73.8) | 50.0 (18.7 - 81.3) | 85.7 (42.1 - 99.6) |
| NPV (95% CI) | 91.7 (73.0 - 99.0) | 90.4 (81.2 - 96.1) | 90.6 (79.3 - 96.9) |

^LRTF locoregional treatment failure, CI confidence interval, PPV positive predictive value, NPV negative predictive value^

Table B. *Low-risk patient group*: diagnostic performance of MRI 6 weeks, 12 weeks, and 24 weeks after completion of chemoradiotherapy to detect locoregional treatment failure (LRTF), with the cutpoint between combined MR tumor regression grade 2 (comrTRG2) and grade 3 (comrTRG3). Clinical outcome (LRTF) served as the reference standard.

|  | ***6 weeks*** | ***12 weeks*** | ***24 weeks*** |
| --- | --- | --- | --- |
| Total (n) | 11 | 41 | 30 |
| LRTF (number) | 0 | 1 | 1 |
| Sensitivity (95 % CI) |  | 0.0 (0.0 - 97.5) | 100.0 (2.5 - 100.0) |
| Specificity (95% CI) |  | 97.5 (86.8 - 99.9) | 100.0 (88.1 - 100.0) |
| PPV (95% CI ) |  | 0.0 (0.0 - 97.5) | 100.0 (2.5 - 100.0) |
| NPV (95% CI) |  | 97.5 (86.8 - 99.9) | 100.0 (88.1 - 100.0) |

^LRTF locoregional treatment failure, CI confidence interval, PPV positive predictive value, NPV negative predictive value^

Table C. *All included patients*: diagnostic performance of MRI 6 weeks, 12 weeks, and 24 weeks after completion of chemoradiotherapy to detect locoregional treatment failure (LRTF), with the cutpoint between combined MR tumor regression grade 1 (comrTRG1) and grade 2 (comrTRG2). Clinical outcome (LRTF) served as the reference standard.

|  | ***6 weeks*** | ***12 weeks*** | ***24 weeks*** |
| --- | --- | --- | --- |
| Total (n) | 45 | 125 | 91 |
| LRTF (n) | 6 | 13 | 12 |
| Sensitivity (95 % CI) | 100.0 (54.1 - 100.0) | 84.6 (54.6 - 98.1) | 75.0 (42.8 - 94.5) |
| Specificity (95% CI) | 30.8 (17.0 - 47.6) | 71.4 (62.1 - 79.6) | 84.0 (75.3 - 90.6) |
| PPV (95% CI) | 18.2 (7.0 - 35.5) | 25.6 (13.5 - 41.2) | 36.0 (18.0 - 57.5) |
| NPV (95% CI) | 100.0 (73.5 - 100.0) | 97.6 (91.5 - 99.7) | 96.6 (90.3 - 99.3) |

^LRTF locoregional treatment failure, CI confidence interval, PPV positive predictive value, NPV negative predictive value^

Table D. *High-risk patient group:* diagnostic performance of MRI 6 weeks, 12 weeks, and 24 weeks after completion of chemoradiotherapy to detect locoregional treatment failure (LRTF), with the cutpoint between combined MR tumor regression grade 1 (comrTRG1) and grade 2 (comrTRG2). Clinical outcome (LRTF) served as the reference standard.

|  | ***6 weeks*** | ***12 weeks*** | ***24 weeks*** |
| --- | --- | --- | --- |
| Total (n) | 34 | 83 | 60 |
| LRTF (n) | 6 | 12 | 11 |
| Sensitivity (95 % CI) | 100.0 (54.1 - 100.0) | 83.3 (51.6 - 97.9) | 72.7 (39.0 - 94.0) |
| Specificity (95% CI) | 25.0 (10.7 - 44.9) | 67.6 (55.5 - 78.2) | 77.6 (63.3 - 88.2) |
| PPV (95% CI) | 22.2 (8.6 - 42.3) | 30.3 (15.6 - 48.7) | 42.1 (20.3 - 66.5) |
| NPV (95% CI) | 100.0 (59.0 - 100.0) | 96.0 (86.3 - 99.5) | 92.7 (80.1 - 98.5) |

^LRTF locoregional treatment failure, CI confidence interval, PPV positive predictive value, NPV negative predictive value^

Table E**.** *Low-risk patient group:* diagnostic performance of MRI 6 weeks, 12 weeks, and 24 weeks after completion of chemoradiotherapy to detect locoregional treatment failure (LRTF), with the cutpoint between combined MR tumor regression grade 1 (comrTRG1) and grade 2 (comrTRG2). Clinical outcome (LRTF) served as the reference standard.

|  | ***6 weeks*** | ***12 weeks*** | ***24 weeks*** |
| --- | --- | --- | --- |
| Total (n) | 11 | 41 | 30 |
| LRTF (n) | 0 | 1 | 1 |
| Sensitivity (95 % CI) |  | 100.0 (2.5 - 100.0) | 100.0 (2.5 - 100.0) |
| Specificity (95% CI) |  | 80.0 (64.4 - 90.9) | 89.7 (72.6 - 97.8) |
| PPV (95% CI) |  | 11.1 (0.3 - 48.2) | 25.0 (0.6 - 80.6) |
| NPV (95% CI) |  | 100.0 (89.1 - 100.0) | 100.0 (86.8 - 100.0) |

^LRTF locoregional treatment failure, CI confidence interval, PPV positive predictive value, NPV negative predictive value^
